# The prognostic value of tumor architecture in patients with upper tract urothelial carcinoma treated with radical nephroureterectomy: A systematic review and meta-analysis

**DOI:** 10.1097/MD.0000000000022176

**Published:** 2020-09-11

**Authors:** Hu Zhao, Lijin Zhang, Bin Wu, Zhenlei Zha, Jun Yuan, Yuefang Jiang, Yejun Feng

**Affiliations:** Department of Urology, Affiliated Jiang-yin Hospital of the Southeast University Medical College, Jiang-yin 214400, China.

**Keywords:** meta-analysis, prognosis, radical nephroureterectomy, tumor architecture, upper tract urinary carcinoma

## Abstract

**Background and purpose:**

There is a lack of consensus regarding the prognostic value of tumor architecture (sessile vs. papillary) in upper tract urothelial carcinoma (UTUC) treated with radical nephroureterectomy (RNU). The aim of the present study was to analyze the current evidence regarding the prognostic role of tumor architecture in patients undergoing RNU for UTUC through a systematic review and meta-analysis.

**Methods:**

According to the Preferred Reporting Items for Systematic Reviews and Meta-analyses (PRISMA) guidelines, a literature search in PubMed, Web of Science, Wanfang, and China National Knowledge Infrastructure (CNKI) databases was performed for citations published prior to February 2020. Cumulative analyses of hazard ratios (HRs) and their corresponding 95% confidence intervals (95% CIs) were conducted for the survival outcomes by Stata 12.0 software.

**Results:**

We retrieved 17 studies (including 8,146 patients) evaluating the effect of tumor architecture on oncologic outcomes in patients treated with RNU. According to our final results, sessile tumor architecture had a significant correlation with worse cancer-specific survival (CSS) (HR = 1.43, 95% CI: 1.31–1.55, *P *< .001), overall survival (OS) (HR = 1.40, 95% CI: 1.24–1.58, *P *< .001), recurrence-free survival (RFS) (HR = 1.43, 95% CI: 1.35–1.53, *P *< .001), and progression-free survival (PFS) (HR = 1.27, 95% CI: 1.11–1.45, *P* = 0.001). The funnel plot test indicated that there was no significant publication bias in the meta-analysis. Besides, the findings of this study were found to be reliable by our sensitivity and subgroup analysis.

**Conclusions:**

Sessile tumor architecture correlates with a significantly worse survival outcome compared with papillary tumor architecture, and it can be used as a valuable biomarker for monitoring prognoses of UTUC patients.

## Abstract 2

Supplemental Digital Content is available in the text

## 1 Introduction

Upper tract urothelial carcinoma (UTUC) is a rare and heterogeneous disease, which involves the renal pelvis and/or the ureter, and it accounts for approximately 5% to 10% of all genitourinary malignancies.^[1,2]^ Although the gold standard for treatment of localized UTUC has been radical nephroureterectomy (RNU) with excision of the bladder cuff, UTUC remains a malignancy with a high potential for local and distant recurrence.^[3,4]^ The reported 5-year recurrence-free survival (RFS) and cancer-specific survival (CSS) rates are 50% to 80% and 70% to 74.4%, respectively.^[5–7]^ Great efforts have been made to improve the understanding of UTUC, but the management for UTUC still remains a big challenge. These unfavorable results highlight the importance of developing a therapeutic strategy to improve the prognosis of UTUC.

Because of the aggressive nature of UTUC, comprehensive recognition of potential prognostic factors is extremely important to improve the therapies. To date, many studies have been conducted to identify significant prognostic factors of UTUC. Pathological stage, tumor location, lymphovascular invasion, tumor necrosis, and concomitant carcinoma in situ were considered important prognostic factors ^[8–11]^. However, these factors have occasionally shown conflicting results. Patients with UTUC in the same stage or grade may experience different comes, which urges us to identify more precise biomarkers to assess the prognosis of UTUC. Actually, urothelial carcinoma with different tumor architectures, is a phenomenon that is well recognized by pathologists ^[12]^. The prognostic value of tumor architecture remains controversial ^[13]^. We hypothesized that sessile tumor architecture may be useful as a prognostic variable to predict the oncological outcomes after RNU. To test this hypothesis, we performed a meta-analysis to verify whether tumor architecture is a prognostic factor influencing the oncological outcome of UTUC through a systematic review and meta-analysis.

## 2 Methods

### 2.1 Search strategy

The electronic databases, PubMed, Web of Science, Wanfang, and China National Knowledge Infrastructure (CNKI) were searched for relevant citations published prior to February 2020. The following search terms were used separately or in combinations: (“upper urinary tract tumor” OR “renal pelvis” OR “ureter”) AND (“radical nephroureterectomy”) AND (“tumor architecture”) AND (“prognosis” OR “clinical outcome” OR “survival”). Reference lists in the previous relevant publications were checked for any other potential studies. The language was restricted to English and Chinese. Two authors independently reviewed the article titles and abstracts according to the Preferred Reporting Items for Systematic Reviews and Meta-analyses (PRISMA) criteria.^[14]^ For all the studies included in this meta-analysis have been published, no ethical approval was needed.

### 2.2 Selection criteria

The **PICOS** (Population, Intervention, Comparator, Outcome and Study design) approach was utilized to define study eligibility: (**P**) patients with UTUC and tumor architecture were pathologically confirmed; (**I**) treatment of RNU; (**C**): sessile tumor architecture and papillary tumor architecture; (**O**): CSS, RFS, overall survival (OS), and progression-free survival (PFS) were the primary endpoints of survival; (**S**) the prognostic value (hazard ratios (HRs) and 95% confidence intervals (95% CIs)) for tumor architecture were reported. Studies were excluded if they met one of the following criteria:

1. studies were not written in English and Chinese;
2. letters, meeting abstracts, commentaries, reviews, or case reports;
3. no data could be extracted from the studies and (or) no sufficient data to estimate the HRs and 95% CIs;
4. When duplicate articles were reported, the most complete and recent studies was selected.

### 2.3 Data extraction

During data extraction, 2 investigators (Z.L.Z. and J.Y.) independently reviewed the articles and extracted the data from the included studies. Any divergences were resolved by consulting the senior author (B.W.). For each selected study, the following items were recorded: publication data (publication year, geographic location, name of the first author, and period of recruitment), baseline clinical characteristics (sample size, median age, gender, treatment received, follow-up period, and oncological outcomes for CSS, OS, RFS, and PFS), tumor pathological characteristics (location of the tumor, tumor multifocality and architecture, tumor stage and grade, and lymph node and surgical margins status).

### 2.4 Quality assessment

The quality of the studies was assessed using the Newcastle-Ottawa Scale (NOS) ^[15]^ for nonrandomized studies, which is recommended by the Cochrane Collaboration. The NOS assesses the quality of studies using a star system based on the following 3 domains: selection of the study groups, comparability of cohorts, and assessment of exposure and outcome. The NOS score ranges from 0 to 9. Studies with scores ≥ 8 were considered to have high quality, those with scores of 6 to 7 were considered to have intermediate quality, and those with scores <6 were considered to have low quality.

### 2.5 Statistical analysis

We conducted a formal meta-analysis to summarize the overall prognostic value of tumor architecture for UTUCs. Due to the observational nature of the included studies, we use pooled log HRs and 95% CIs for the oncologic survival outcomes of CSS, OS, RFS, and PFS. The cumulative effects of tumor architecture were evaluated by the inverse variance method. An observed HR > 1 indicated a worse survival for patients with sessile tumor architecture expression. Chi-squared test and the I^2^ statistic were used to assess the heterogeneity among studies. *P* value <.1 or an *I*^2^ > 50% suggested the presence of significant between-study heterogeneity. Therefore, we calculated pooled HRs using the random-effects (RE) model. Alternatively, when no significant heterogeneity was found, we used the fixed-effect (FE) model to perform cumulative analyses. To assess the risk of publication bias, we used Egger linear regression and funnel plots for outcomes in this meta-analysis. Potential sources of heterogeneity were identified using subgroup analyses. Sensitivity analysis was performed by omitting each study involved in the meta-analysis, and then evaluating the stability of results. Statistical analyses were performed using Stata12.0 statistical software (Stata Corp, College Station, TX, US). All *P* values were two-sided and *P* value < .05 was considered statistically significant.

## 3 Results

### 3.1 Search results

**Figure 1:**
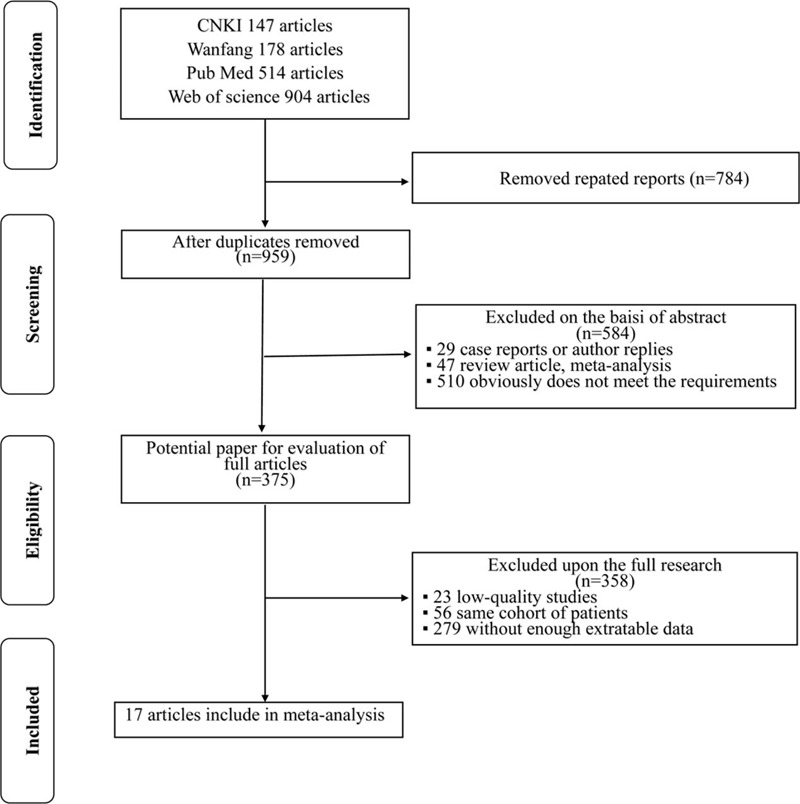
Preferred reporting items for systematic reviews and meta-analyses (PRISMA) flow chart of literature search and selection process.

Figure 1 shows a detailed flow chart of our selection process. Following an initial electronic search, a total of 1743 possibly eligible articles were identified in this meta-analysis. After screening the titles and abstracts, we removed 1368 duplicate or irrelevant articles. Consequently, the remaining 375 studies were considered potentially relevant articles for further full-text review. After punctilious reading, 358 studies were excluded; of these, 279 studies were excluded because they did not provide sufficient data, 56 articles were excluded as the same participants were included in other studies, and 23 articles were excluded as there were of low quality. Finally, 17 studies^[12,16–31]^ published from 2014 to 2019, which met all of the inclusion and exclusion criteria, were enrolled in this meta-analysis.

### 3.2 Characteristics of the studies

The main characteristics of the 17 studies are summarized in Tables 1 and 2. Briefly, a total of 8146 participants (ranging from 100 to 1086) were included in our meta-analysis. All studies had a retrospective study design. All patients in these studies had pathologically confirmed UTUC with different tumor architectures and had received RNU. Of the 17 studies, 9 were conducted in China, 3 in Japan, 2 in Korea, and 3 at international multi-centers. Among the studies, 15 studies were performed to analyze CSS, 10 studies were conducted to investigate RFS, 9 studies were conducted to investigate OS, and 6 studies reported PFS. All included articles were published in English. The NOS showed all studies were of high quality, with NOS score ≥7 (Supplementary Table 1).

**Table 1:**
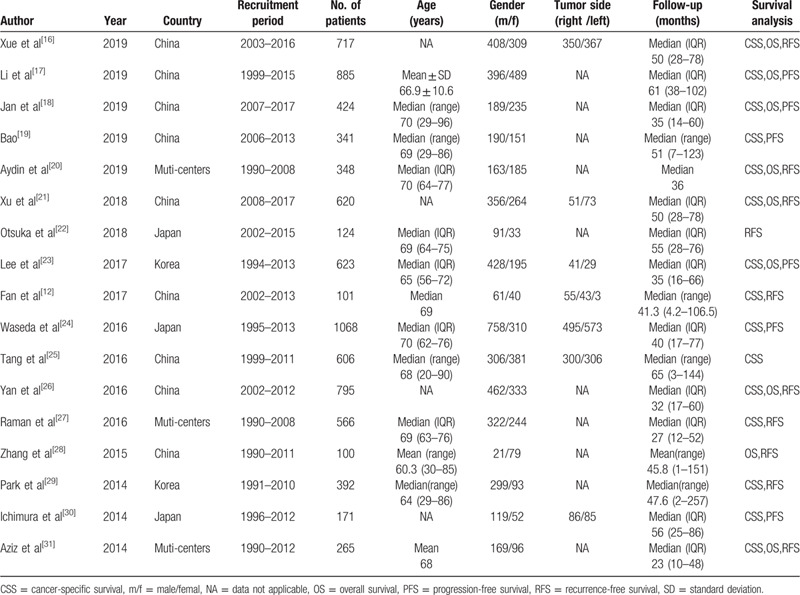
The baseline clinical characteristics of the studies included in this meta-analysis.

**Table 2:**
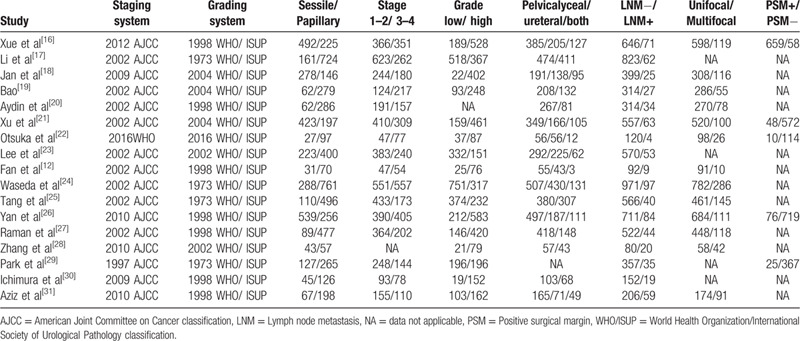
The main oncology characteristics of the studies included in this meta-analysis.

### 3.3 Meta-analysis

**Figure 2:**
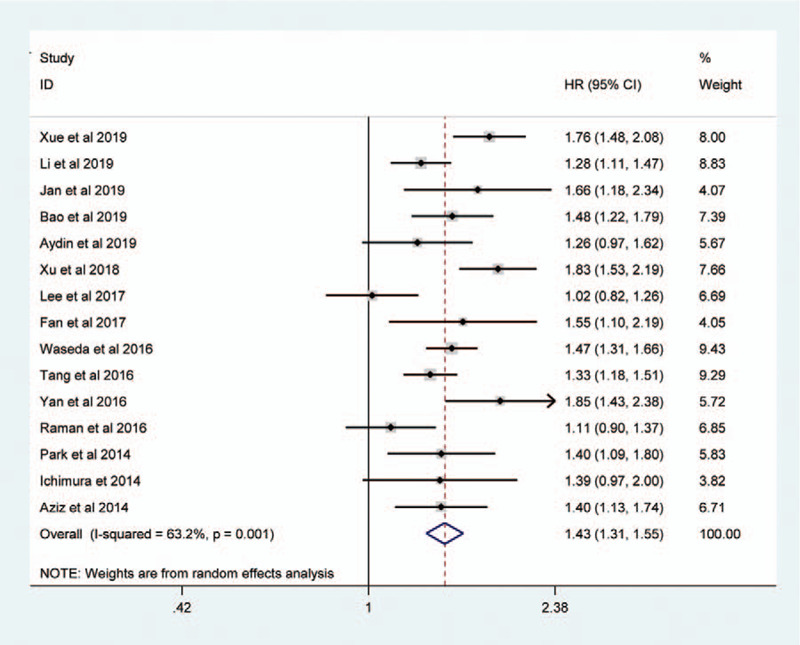
Forest plots showing the association between tumor architecture (sessile vs papillary) and CSS.

**Figure 3:**
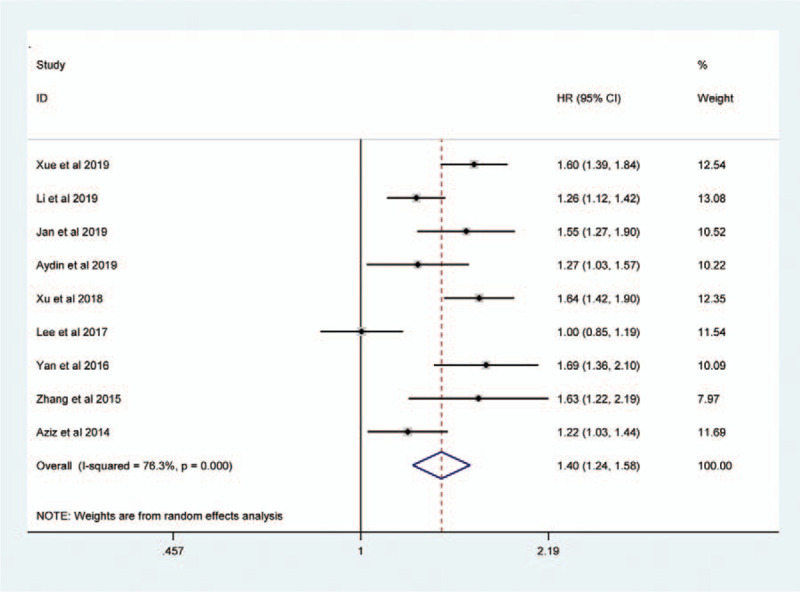
Forest plots assessing the correlation of tumor architecture (sessile vs papillary) with OS.

**Figure 4:**
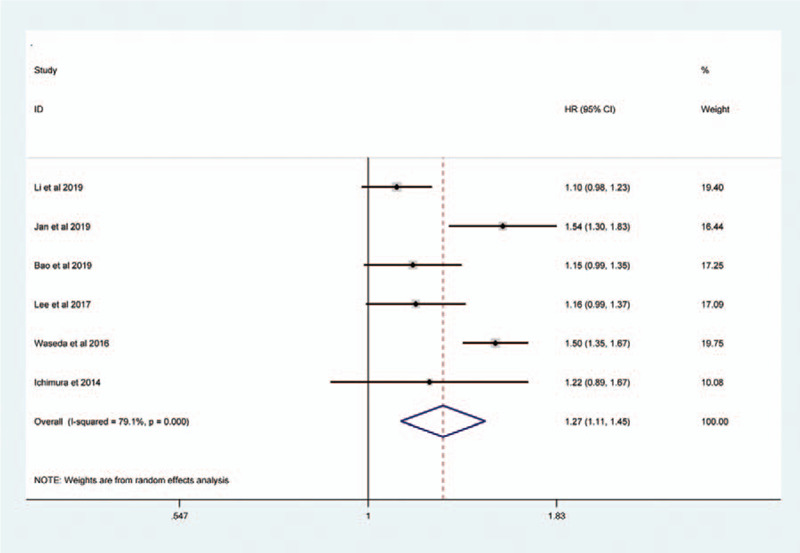
Forest plots showing the association between tumor architecture (sessile vs papillary) and PFS.

**Figure 5:**
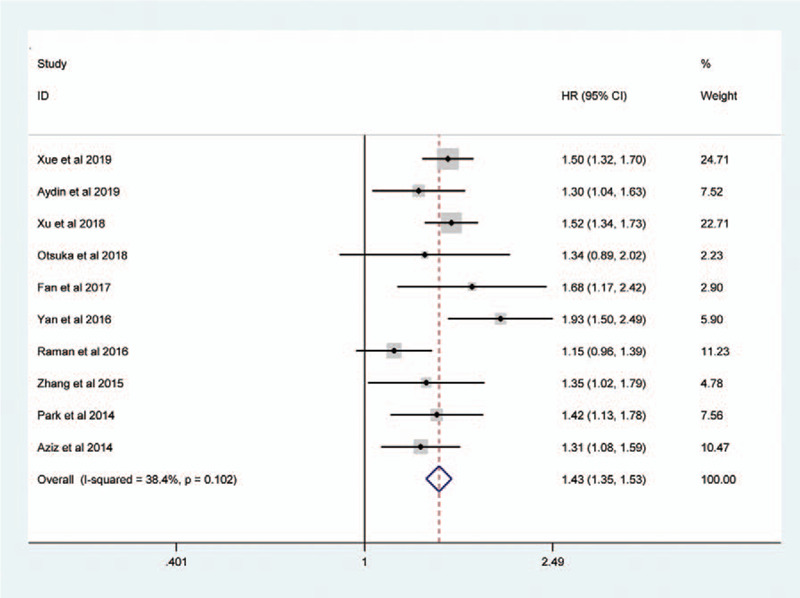
Forest plots assessing the correlation of tumor architecture (sessile vs papillary) with RFS.

Sessile tumor architecture was reported in 3067 of 8146 patients (36.7%). The pooled HR across these studies indicated that sessile tumor architecture of UTUC was associated with worse CSS (HR = 1.43, 95% CI: 1.31–1.55, *P *< .001, Fig. 2), OS (HR = 1.40, 95% CI: 1.24–1.58, *P *< .001, Figure 3), and PFS (HR = 1.27, 95% CI: 1.11–1.45, *P* = 0.001, Figure 4). Significant heterogeneity was observed in the CSS (Chi^*2*^ = 38, *I*^*2*^ = 63.2%), OS (Chi^*2*^ = 33.7, *I*^*2*^ = 76.3%), and PFS (Chi^*2*^ = 23.9, *I*^*2*^ = 79.1%); hence we used the RE model. Besides, the forest plot showed that sessile tumor architecture was significantly associated with poor RFS (HR = 1.43, 95% CI: 1.35–1.53, *P *< .001, Fig. 5). The *I*^*2*^ test (Chi^2^ = 14.6, *I*^*2*^ = 38.4%) showed moderate heterogeneity; therefore, the FE model was adopted to calculate the pooled HR. To explore the heterogeneity, subgroup analysis under the geographical region (Asia vs non-Asian), year of publication (≥2016 vs <2016), TNM stage (T3+T4%) (≥ 50 vs < 50), tumor grade (G2+G3%) (≥70 vs <70), no. of patients (≥ 500 vs <500), and median follow-up (≥40 months vs <40 months) was performed. Pooled HRs were significantly and consistently higher than 1 in the subgroup meta-analysis. The observed heterogeneity was reduced significantly in some subgroup models, such as Geographical region in non-Asian areas, No. of patients <500, Stage (T3+T4%) 50%, and Grade (G3+G4%) ≥ 70% (Table 3).

**Table 3:**
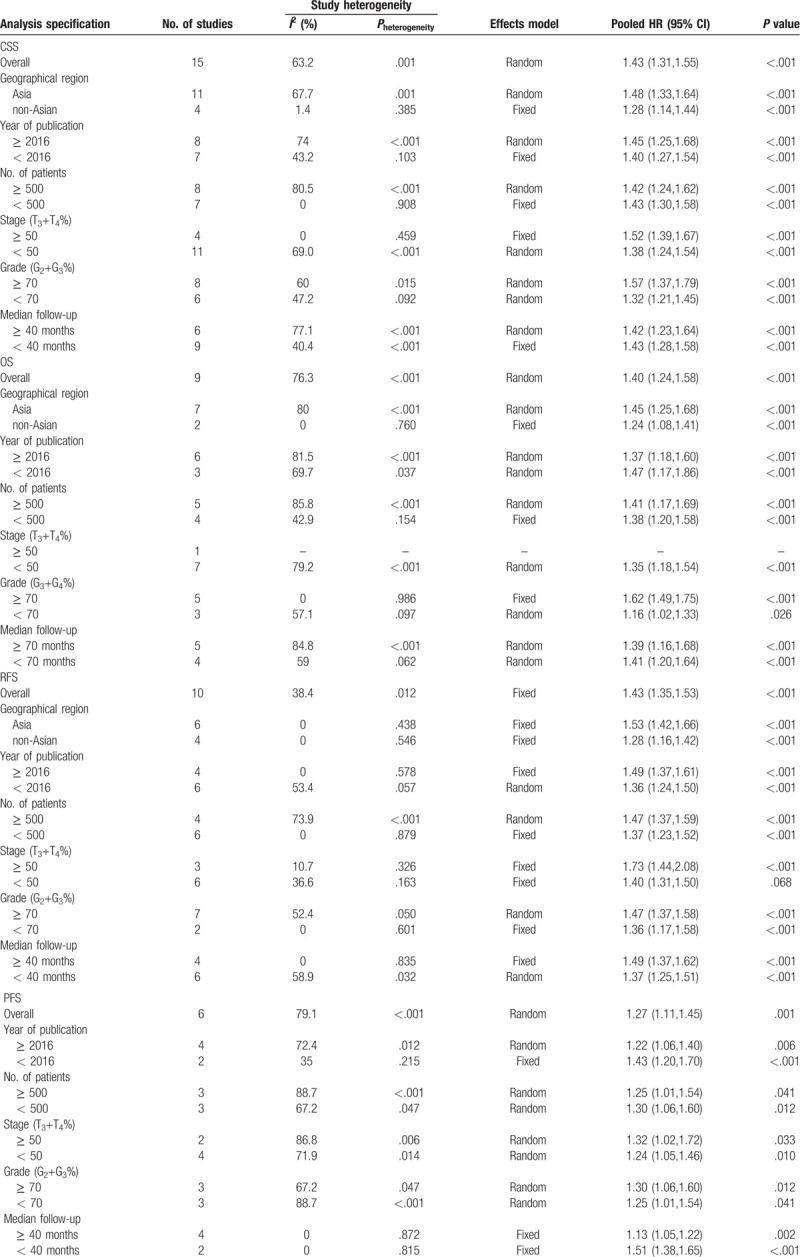
Summary and subgroup analysis of pooled HRs for the included studies.

### 3.4 Sensitivity analysis

Each single study was omitted to estimate the influence of individual data on the pooled HR. As shown in Supplementary Fig. S1, the pooled HR for CSS ranged from 1.39 (95% CI: 1.28–1.52) to 1.46 (95% CI: 1.35–1.58) (Supplementary Fig. S1a), the pooled HR for OS ranged from 1.37 (95% CI: 1.21–1.55) to 1.46 (95% CI: 1.32–1.61) (Supplementary Fig. S1b), the pooled HR for RFS ranged from 1.41 (95% CI: 1.32–1.50) to 1.47 (95% CI: 1.38–1.57) (Supplementary Fig. S1c), and the pooled HR for PFS ranged from 1.22 (95% CI: 1.07–1.38) to 1.32 (95% CI: 1.15–1.51) (Supplementary Fig. S1d). The results of the sensitivity analysis showed that no study had a significant effect on the observed pooled HR, indicating the reliability of our findings.

### 3.5 Publication bias

**Figure 6:**
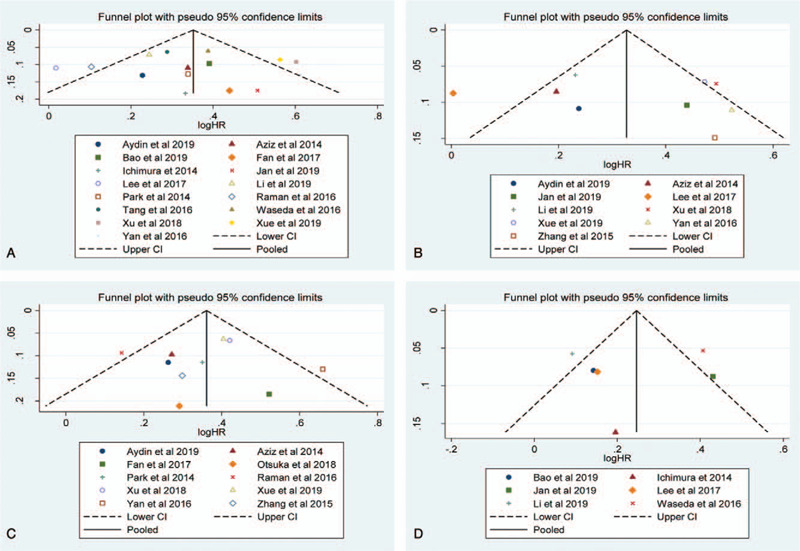
Funnel plots evaluating potential publication bias regarding (a) CSS, (c) OS, (c) RFS, and (d) PFS.

Publication bias was detected using a funnel plot and Eggers test. As presented in Figure 6, the shapes of the funnel plots indicated that there was no evident asymmetry. The Eggers test for CSS (*P*-Egger = .828, Fig. 6A), OS (*P*-Egger = .689, Fig. 6B), RFS (*P*-Egger = .903, Fig. 6C), and PFS (*P*-Egger = .830, Fig. 6D) did not show any evidence of publication bias in our meta-analysis.

## 4 Discussion

Compared to bladder cancers, UTUCs are usually more invasive tumors at diagnosis and are significantly associated with high recurrence and progression rates.^[32]^ Despite efforts, little is known about the natural history and impact of prognostic variables in UTUC. Potential prognostic factors include baseline clinical variables (age,^[33]^ body mass index,^[34]^ and gender^[35]^) and pathologic features obtained after RNU, such as pathologic stage, lymph node metastasis, and tumor grade, seem to be well established.^[36,37]^ However, the accuracy of these prognostic factors is not sufficient for clinical risk stratification. We hypothesized that sessile tumor architecture and papillary tumor architecture may not be the same disease in terms of invasion and prognosis. A number of studies have examined the prognostic role of tumor architecture in UTUC; nevertheless, the coherence and importance of the prognostic value of tumor architecture still need to be explored. Most previous studies were limited to an insufficient number of patients for performing the systematic analyses for the prognostic value of tumor architecture. Before tumor architecture can be integrated into clinical decision making, it needs to be validated in an independent data set. Therefore, the aim of the current study was to identify the prognostic significance of tumor architecture in UTUC patients after radical surgery.

Accumulating evidence indicates that tumor architecture may indicate a more advanced stage and it may be associated with more aggressive oncological behavior in UTUC patients. Fan et al^[12]^ and Margulis et al^[38]^ showed that sessile tumor architecture was significantly associated with the risk of disease recurrence and it was proved to be a reliable prognostic factor in patients with UTUC. Remzi et al ^[13]^ provided evidence that sessile tumor architecture was associated with more aggressive behavior and was an independent risk factor for tumor recurrence and CSS after RNU. Fritsche et al ^[39]^ confirmed the strong independent prognostic value of tumor architecture in a large, multicenter UTUC cohort of 754 patients. Remzi et al and Fritsche et al recommended that tumor architecture should be routinely reported by pathologists, and it should be identified to help in clinical decision-making regarding the postoperative follow-up and treatment protocol.

Potential reasons underlying the worse outcomes in patients with sessile tumor architecture may be related to more aggressive biologic features of tumors or a delay in diagnosis or treatment ^[40]^. However, some observational studies failed to show the impact of tumor architecture on UTUC outcomes. For instance, Li et al^[17]^ found no association between sessile tumor architecture and RFS in a multivariate analysis model. Also, Park et al^[29]^ did not identify tumor architecture as a significant risk factor for CSS and RFS in pT3 UTUC patients who underwent RNU. Since meta-analysis can integrate the findings on specific topics, we performed a large collection of analysis to provide a comprehensive summary based on the published literatures to report the data for tumor architecture and their effects on UTUC prognosis.

To the best of our knowledge, the present study is the first meta-analysis of the association between tumor architecture and oncological outcomes in UTUC patients. In the current study, we found that sessile tumor architecture was present in 36.7% of patients treated with RNU. Consistent with previous publications, sessile tumor architecture was associated with poor outcomes in terms of CSS (HR = 1.43, *P *< .001), OS (HR = 1.40, *P *< .001), RFS (HR = 1.43, *P *< .001), and PFS (HR = 1.27, *P* = .001) in UTUC patients. To identify the source of heterogeneity, we performed a subgroup analysis that was stratified by several potential influencing factors. Interestingly, when stratified according to ethnicity, significantly increased risks were identified among non-Asian patients for CSS, OS, and RFS. These findings indicate that sessile tumor architecture for UTUC prognosis may have an ethnic difference. On the other hand, the subgroup analysis revealed that the association of sessile tumor architecture with worse survival was stronger in higher tumor stages and grades, which were in accordance with conclusions from other studies^[12,13,39]^. Taking the above results together, we concluded that sessile tumor architecture expression predicted poor prognosis and sessile tumor architecture, and patients with UTUC may need a closer follow-up.

There are several limitations in this study that need to be addressed. First, most populations included in this meta-analysis were of Asian ethnicity; thus, ethnicity bias may exist and the conclusion may not be the same in other races. Therefore, additional populations from other ethnicities are required to further validate the ethnic difference in the effect of tumor architecture on UTUC risk. Second, all enrolled studies were retrospective in nature, and information and selection biases cannot be excluded. Third, although we searched the relevant Chinese literature, this study was limited to articles published in English, which might contribute to selection bias and publication bias. Finally, obvious heterogeneity among studies was observed in several analyses. To solve this problem, we conducted a subgroup analysis to explore the heterogeneity sources, and the results showed that between-study heterogeneity was possibly associated with the source of patients and biological features of the tumor. Therefore, the conclusion should be considered cautiously.

In spite of these potential limitations, this meta-analysis has its own advantages and strengths. First, to guarantee the study quality in our meta-analysis, we used the NOS to evaluate the methodological quality of each study. As a result, all articles included in the final analysis were of high quality. Second, we manually searched the reference lists of the included studies to collect more eligible articles, upon the extensive search strategy, as much as possible. Third, Eggers test was performed to detect publication bias, which is more reliable than visual observation of funnel plots. Furthermore, the sample size in this meta-analysis was larger than any individual study; therefore, providing more reliable results. Thus, the present study may provide a more powerful conclusion on the relationship between sessile tumor architecture and UTUC.

## 5 Conclusion

Our investigations suggest that sessile tumor architecture predicted a poor CSS, OS, RFS, and PFS in UTUC patients. These findings infer that sessile tumor architecture is a potential adverse prognostic marker for patients with UTUC. Integration of tumor architecture with other factors may help in risk stratification and individualized treatment of patients with UTUC after RNU. Considering the limitations mentioned above, further well-designed studies with different ethnicities are warranted to confirm our results.

## Author contributions

HZ: Project development, Manuscript writing; LJZ: Project development, Data Management, Manuscript editing; BW: Data Collection; ZLZ: Data Collection; JY: Data Collection; YFJ: Data analysis, Data Management; YJF: Data analysis, Data Management. All authors have read and approved the manuscript.

## Supplementary Material

Supplemental Digital Content

## Supplementary Material

Supplemental Digital Content
