# Supplementary material for: The prognostic value of tumor architecture in patients with upper tract urothelial carcinoma treated with radical nephroureterectomy: A systematic review and meta-analysis
Source: Medicine (Baltimore). 2020 Sep 11;99(37):e22176. doi: 10.1097/MD.0000000000022176 (PMC7489711; doi:10.1097/MD.0000000000022176)
Supplement: Supplemental Digital Content [file medi-99-e22176-s001.docx]

**Table S1.** Quality assessment of the studies included in this meta-analysis

| **Study** | **Representativeness of the exposed cohort** | **Selection of the unexposed cohort** | **Ascertainment of exposure** | **Outcome of interest not present at start of study** | **Control for important factor or additional factor** | **Outcome assessment** | **Follow-up long enough for outcomes to occur** | **Adequacy of follow-up of cohort** | **Total quality scores** |
| --- | --- | --- | --- | --- | --- | --- | --- | --- | --- |
| Xue et al.[[15](#_ENREF_15)] | ★ | ★ | ★ | ★ | ★★ | ★ | ★ | ★ | 9 |
| Li et al.[[16](#_ENREF_16)] | ★ | ★ | ★ | ★ | ★★ | ★ | ★ | ★ | 9 |
| Jan et al.[[17](#_ENREF_17)] | ★ | ★ | ★ | ★ | ★★ | ★ | ★ | ★ | 9 |
| Bao.[[18](#_ENREF_18)] | ★ | ★ | ★ | ★ | ★★ | ★ | ★ | ★ | 9 |
| Aydin et al.[[19](#_ENREF_19)] | ★ | ★ | ★ | ★ | ★★ | ★ | ★ | ★ | 9 |
| Xu et al.[[20](#_ENREF_20)] | ★ | ★ | ★ | ★ | ★★ | ★ | ★ | ★ | 9 |
| Otsuka et al.[[21](#_ENREF_21)] | ★ | ★ | ★ | ★ | ★ | ★ | ★ | ★ | 8 |
| Lee et al.[[22](#_ENREF_22)] | ★ | ★ | ★ | ★ | ★★ | ★ | ★ | ★ | 9 |
| Fan et al.[[11](#_ENREF_11)] | ★ | ★ | ★ | ★ | ★ | ★ | ★ | ★ | 8 |
| Waseda et al.[[23](#_ENREF_23)] | ★ | ★ | ★ | ★ | ★★ | ★ | ★ | ★ | 9 |
| Tang et al.[[24](#_ENREF_24)] | ★ | ★ | ★ | ★ | ★★ | ★ | ★ | ★ | 9 |
| Yan et al.[[25](#_ENREF_25)] | ★ | ★ | ★ | ★ | ★★ | ★ | ★ | ★ | 9 |
| Raman et al.[[26](#_ENREF_26)] | ★ | ★ | ★ | ★ | ★★ | ★ | ★ | ★ | 9 |
| Zhang et al.[[27](#_ENREF_27)] | ★ | ★ | ★ | ★ | ★★ | ★ | ★ | ★ | 9 |
| Park et al.[[28](#_ENREF_28)] | ★ | ★ | ★ | ★ | ★★ | ★ | ★ | ★ | 9 |
| Ichimura et al.[[29](#_ENREF_29)] | ★ | ★ | ★ | ★ | ★ | ★ | ★ | ★ | 8 |
| Aziz et al.[[30](#_ENREF_30)] | ★ | ★ | ★ | ★ | ★ | ★ | ★ | ★ | 8 |
