# Supplementary material for: The prognostic value of tumor architecture in patients with upper tract urothelial carcinoma treated with radical nephroureterectomy: A systematic review and meta-analysis
Source: Medicine (Baltimore). 2020 Sep 11;99(37):e22176. doi: 10.1097/MD.0000000000022176 (PMC7489711; doi:10.1097/MD.0000000000022176)

**Supplementary Figure S1.** Sensitivity analysis in this meta-analysis. (a) Sensitivity analysis for CSS; (b) sensitivity analysis for OS; (c) sensitivity analysis for RFS; and (d) sensitivity analysis for PFS.


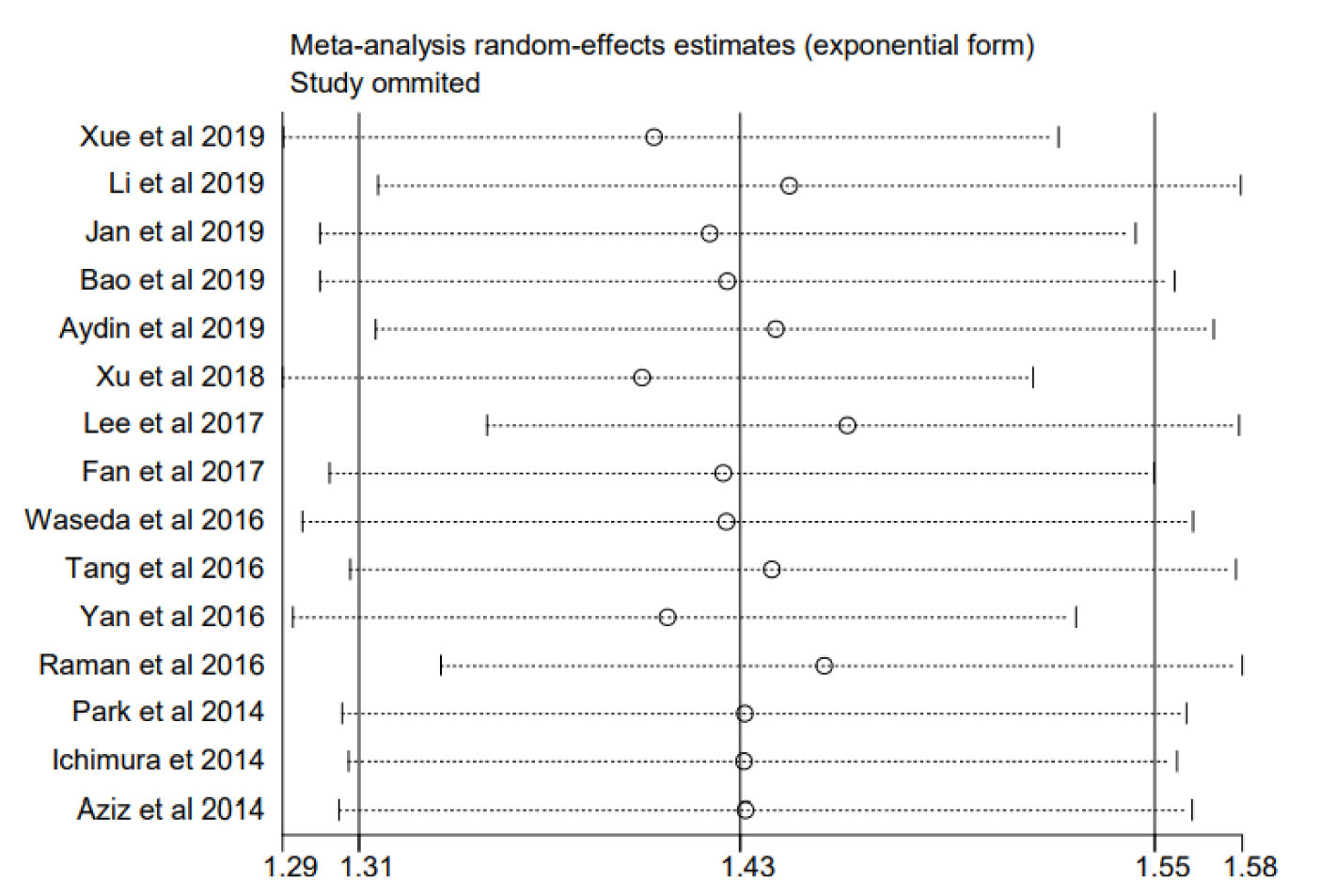


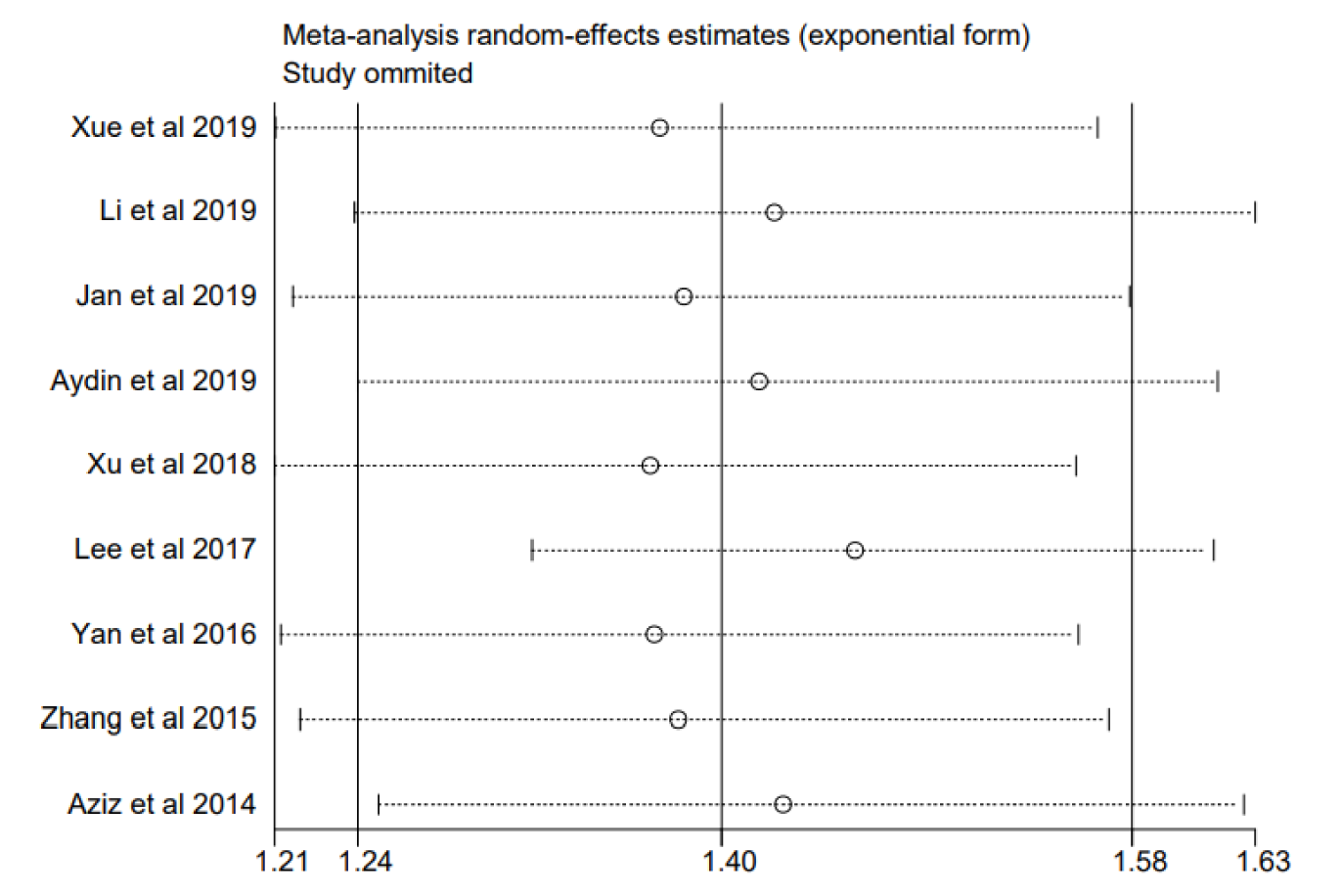


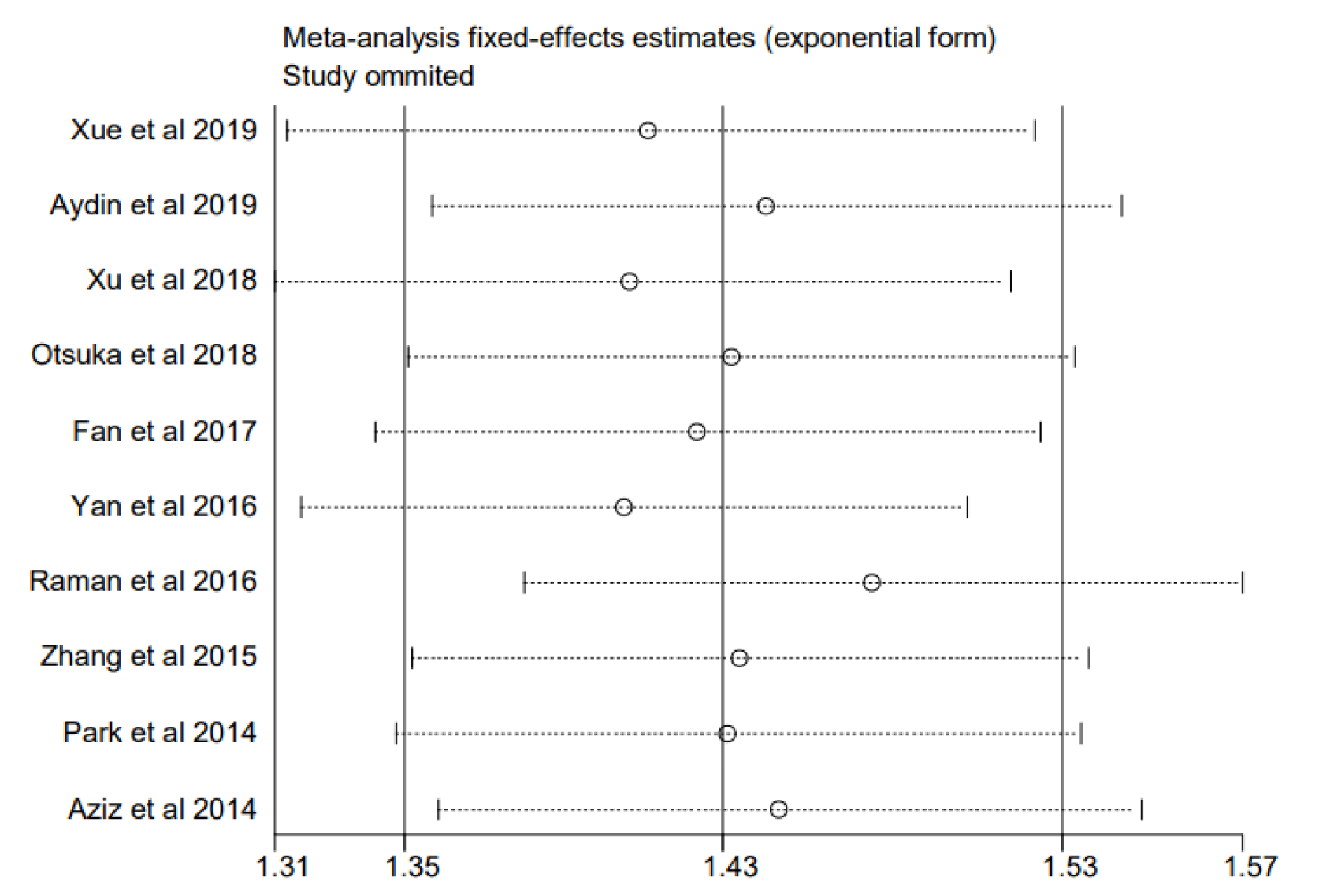


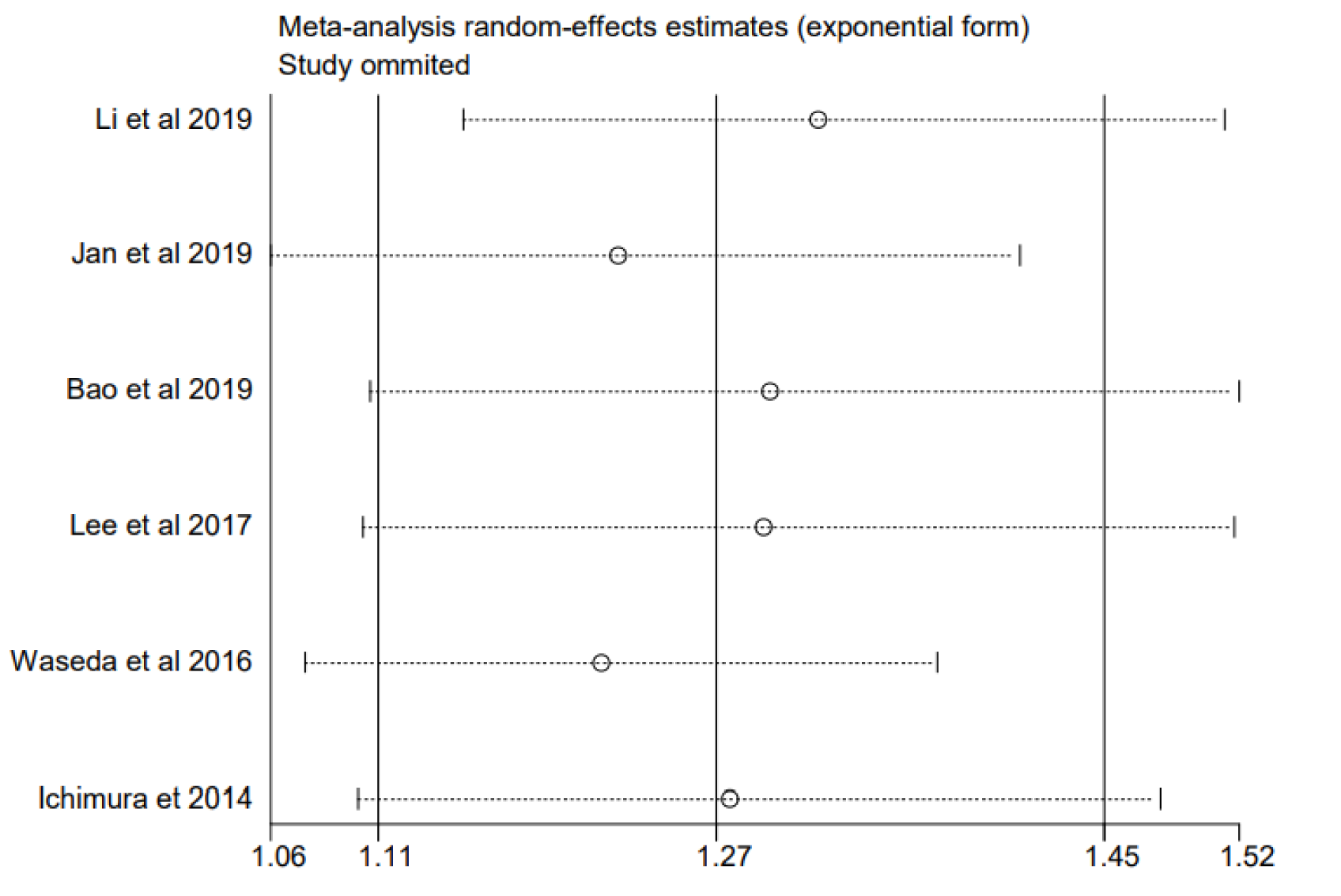

Supplement: Supplemental Digital Content [file medi-99-e22176-s002.docx]
